# Home environmental change for child injury prevention in Nepal: A qualitative study

**DOI:** 10.1177/13674935211052156

**Published:** 2021-11-29

**Authors:** Santosh Bhatta, Julie Mytton, Toity Deave

**Affiliations:** 1 Research Associate, Faculty of Health and Applied Sciences, 1981 University of the West of England, Bristol, UK; 2 Professor of Child Health, Faculty of Health and Applied Sciences, 1981 University of the West of England, Bristol, UK; 3 Associate Professor for Family & Child Health, Faculty of Health and Applied Sciences, 1981 University of the West of England, Bristol, UK

**Keywords:** child, wounds and injuries, built environment, safety, Nepal

## Abstract

In Nepal, unintentional home injury is a leading reason for death and disability among pre-school children. However, there is a lack of evidence to inform culturally appropriate interventions to reduce home injuries. This study explored the potential for home environmental change at a community level to prevent unintentional home injury in children and identified the barriers to, and facilitators of, such changes. Focus groups were conducted in the Nepali language with mothers, fathers, teachers, school students and community health volunteers in rural areas of Makwanpur district in Nepal. The discussions were audio-recorded, transcribed, translated into English and analysed thematically. NVivo software was used to support coding and identification of themes. Five focus groups, involving forty-seven participants, were completed leading to the development of four themes. Overall, the findings highlight that community people perceive injuries to be a normal part of childhood and, therefore, few prevention measures were considered. Parents were, however, able to identify ways to change their environment that made it safer. Changes included removing hazards or adding safety equipment, adapting the home or restricting access to potential hazards. Barriers to implementation included limited awareness about injury hazards and risk management, poor quality housing and financial constraint. Facilitators included raising community awareness, acquiring resources and financial support and involving the family and community. Development of interventions to prevent injuries at home in pre-school children should reflect local context and culture; this is best achieved through engagement with parents.

## Introduction

Worldwide, unintentional home injuries are a major cause of preventable death and serious disability among children under the age of 5 years (Sengoelge et al., 2011). These deaths have a huge impact on families and society, particularly in low- and middle-income countries (LMICs), where injury-related mortality among young children is four to six times higher than in high-income countries (HICs) (Peden et al., 2008). Low-income communities are more likely to live in environments with a greater number of hazards (Peden et al., 2008). Children’s exposure to those hazards is likely to increase their risk of injury (Bartlett, 2002). More than 50% of injuries among pre-school children occur in the home environment where they spend most of their time (Fatmi et al., 2009; Hyder et al., 2009).

In many LMICs, preventive safety measures are limited not only by the economic situation but also by a cultural tendency to view injury as a consequence of an unavoidable accident (Bartlett, 2002). In fact, like with many diseases, unintentional injuries are caused by events that have predictable, and therefore preventable, outcomes (Davis and Pless, 2001). Poorer families are less likely to use or have access to safety information and are exposed to a larger range of hazards in the home environment, thus, increasing the likelihood of injury in their children (Ruiz-Casares, 2009; Ward and Viner, 2017). The common home hazards for children in low-income settings are poor housing infrastructure, lack of barriers to cooking or washing areas, open fires and paraffin stoves that are accessible to children, lack of safe storage for harmful substances, unprotected balconies, stairs and open water reservoirs (Balan and Lingam, 2012; Hyder et al., 2008; Bhatta et al., 2020).

Qualitative studies can support child injury prevention through exploration of a range of factors, including: perception of the causes of injuries and prevention measures (Butchart et al., 2000; Mashreky et al., 2009; Rahman et al., 2008), parents’ knowledge, attitude and beliefs related to childhood injuries (Morrongiello and Dayler, 1996), mothers’ beliefs about parenting and injury prevention (Murphy, 2001) and parents’ perception, attitude and behaviours towards child safety (Vincenten et al., 2005). Recognising and applying a combination of preventive approaches such as increasing public awareness, behavioural change programmes, environmental changes and legislative changes for those who are most vulnerable to injury could potentially prevent injury and reduce injury inequality worldwide (Watson and Errington, 2016; Peden et al., 2008).

People’s perceptions of injury risk and prevention measures differ depending on their professional, social and personal backgrounds (Rothe, 2000; Stone and Morris, 2010). Therefore, to develop and deliver appropriate, feasible and acceptable interventions it is important to understand the perceptions and values of those to whom interventions are targeted (Dowd, 1999; Roberts, 1997). Qualitative studies are considered to be the best way to understand people’s perceptions or experiences, attitudes, beliefs and the meaning of their experiences (Creswell and Creswell, 2017). In Nepal, there are limited data available from qualitative studies to understand injuries that occur to children in the home, their causes and potential preventive measures; this study aims to fill this research gap.

## Aim

To explore the potential for home environmental change at a community level to prevent unintentional home injuries to pre-school children and identify the barriers to, and facilitators of, such changes.

## Methods

### Study design

This qualitative study employed focus groups (Bowling, 2014; Creswell and Creswell, 2017) to capture participants’ views and discuss their ideas about home environmental change for child injury prevention in rural Nepalese communities.

### Sample and settings

Purposive sampling was used to elicit a broad range of insights into injury risks and their management. Inclusion criteria: individuals representing the rural community and were either directly or indirectly related to child health. We recruited individuals from five distinct groups: mothers, Female Community Health Volunteers (FCHVs), Early Childhood Education and Development (ECED) teachers, fathers and school students. The FCHVs are the frontline practitioners who work directly with parents to improve maternal and child health in Nepal (Khatri et al., 2017). Individuals in each group had similar backgrounds, but individuals between groups differed in terms of occupation, age, education and living area. Each focus group had only one type of carer. For example, in the mothers’ group, all of the individuals were mothers.

This was an exploratory study designed to complement the findings of a household survey undertaken by the author (Bhatta et al., 2020). In conjunction with the survey results, these five focus groups were deemed adequate to provide a comprehensive picture of home injury hazards in rural Nepal. Mother and Infant Research Activities (MIRA), a non-governmental organisation facilitated the recruitment of participants. (http://www.mira.org.np/mira/). Potential participants were mostly approached face-to-face and in some cases *via* a phone call. Study information was provided to the participants prior to the focus group.

The study was carried out during March and April 2015 in three different topographical regions of Makwanpur district. All the focus groups participants were from rural areas in each selected region. The focus group with mothers were conducted in an open area designated for the monthly meetings of the mothers’ group. These Mother’s Groups are an established practice across the country. In rural Nepal, groups of mothers meet monthly to discuss health issues, facilitated by a leader and a FCHV. The focus group with the FCHVs was conducted in their usual meeting room at the local-health post. A health-post is the first institutional contact point for basic health services and referral centre for FCHVs. The focus group with ECED teachers was conducted in a community meeting centre, fathers participated in a meeting room of a local school and school students in a meeting room at the higher secondary school.

### Data collection

A topic guide was developed and used across all five focus groups. The topic guide included three issues to help structure the group discussion: home injury and associated hazards, potential home environmental changes and barriers to and facilitators of such changes. Guiding questions based on available literature (Munro et al., 2006; Pant et al., 2014) and consultation with the research team were developed to explore these three issues.

At the start of each focus group, the researcher informed participants about the study’s objectives and assured the participants that the discussions would be kept confidential. The researcher provided each participant with the opportunity to ask questions and to consider participation. Those who agreed to participate completed and signed a consent form. The researcher also informed the participants that they could agree or disagree with one another in order to gain a better understanding of how the group thought about the issue in terms of beliefs, experiences and practices.

A researcher (SB) and two MIRA staff, recruited and trained to facilitate the data collection, conducted all the focus groups. All the discussions took place face-to-face and were audio-recorded. Notes were taken to identify which participant was speaking and to record their body language. SB led the focus groups with MIRA staff taking notes. The researcher and note-takers discussed the notes immediately following each focus group to ensure agreement on key issues.

### Data analysis

The MIRA staff transcribed all the audio-recordings verbatim in Nepali and then translated the data into English. Data were pseudonymised during transcription. SB listened to each audio-recording with the translated text to ensure the original meaning of the statements was retained during transcription and translation. According to the guidelines provided by Braun and Clarke (2006), thematic analysis was applied to identify, analyse and report repeated patterns of meaning that were drawn inductively from the data.

The NVivo Qualitative Data Analysis Software, Version 12 (NVIVO, 2018), was used for the initial data coding. To assist in establishing credibility of the findings (MacPhail et al., 2016), TD and JM each read two different transcripts independently to help develop a coding framework. The coding framework was then applied across the transcripts. Any contradictory results in terms of themes and codes were added or removed by consensus. Codes were arranged and collated systematically to identify candidate themes. Pseudonyms have been used when reporting participant’s quotes (e.g. Rama and Mothers focus group).

### Ethics approval

Ethical approval was obtained from the Nepal Health Research Council in Kathmandu, Nepal (Registration no. 273/2014) and from the Research Ethics Committee of the Faculty of Health and Social Care, at the University of the West of England, Bristol, UK (Reference no. HAS.15.01.88).

### Findings

Five focus groups, with a total of forty-seven participants, were completed across three different topographical regions. Discussions lasted between 40 and 75 min. Table 1 shows the characteristics of the participants.

**Table 1.** Characteristics of study participants.

Focus group	Participants	Age range of participants	Number of participants	Educational attainment of participants	Topographical region where group held
1	Mothers	24–40 years	9 females	2 – primary school	High hill
				2 – read and write only	
				5 – illiterate	
2	*FCHVs	21–52 years	8 females	3 – lower secondary school	Mid hill
				1 – higher secondary school	
				4 – primary school	
3	**ECED teachers	20–38 years	9 females	8 – secondary school	Mid hill
				1 – lower secondary school	
4	Fathers	27–45 years	12 males	1 – lower secondary school	Low land
				11 – primary school	
5	School students	13–16 years	9 (4 boys and 5 girls)	All studying in grade 9	Mid hill

*FCHVs = Female Community Health Volunteers, **ECED = Early Childhood Education and Development.

Four themes were identified by analysing the focus group data: perception that child injuries are normal; limited awareness of injury hazards and risks; motivation and affordability to reduce hazards; cultural practices and shared responsibilities. Participants’ quotes are used to illustrate each theme.

### Perception that child injuries are normal

Across all the groups, the participants expressed their perceptions of child injuries in different ways; This could be due to the fact that they come from very different backgrounds and have had very different experiences. Most, however, stated that childhood injury is a daily thing and part of child development.

*Cuts with a sickle are common among children because they see their parents or other adult family members using them. There are no major injuries, just minor ones. What can we do when they play and cut? [Rama, Mothers focus group]*

During a discussion with the teachers’ group, one participant said that no parent wanted their children to get ill or suffer from any kind of harm. Although the carelessness was not deliberate, unintended injuries occurred. The teacher also mentioned that, despite her best efforts, injury to children still occurred. Other teachers agreed that avoiding injury in young children was a challenging task due to the unexpected nature of the children’s behaviour.

*“Preventing injury in children, particularly those under the age of five, is extremely difficult. When they cross the age of 5 years, things will be much easier.” [Gita, Teachers focus group]*

In general, participants did not see minor injuries as a cause for concern, and therefore were generally disregarded by parents and caregivers. Only serious injuries seemed to get notice. The overarching conclusion that came from all group discussions was that child injury is a prevalent issue in rural areas. Participants said that falls, drowning, fire or burns, poisoning, animal or insect bites and suffocation were all well-known causes of death in their communities. The Supplementary Material contains incidents resulting in death as a result of various kinds of injuries reported by participants (Supplementary Table S1).

### Limited awareness of injury hazards and risks

Most of the participants’ knowledge about home hazards was from experience. They were less aware of potential hazards and children safety measures. Common home injury hazards identified during the focus groups are summarised in the Supplementary Table S1. Mothers appeared to be the least aware of the risks of injury. One mother expressed her level of knowledge about hazards as follows:

*“We have limited knowledge about this issue. Now that you’re talking about it, we are able to give you some examples from our own home and neighbourhood. Other than that, we do not have any other sources of information, and we don’t know much.” [Sita, Mothers focus group]*

Participants in all of the focus groups reported that this was their first-time discussing home safety. Most parents and guardians in their communities, according to health volunteers, had not received any information about injury risks in the home. Parents could have made changes in their homes if they had known.

*“They [parents] are unaware of and do not anticipate injury risks. They might be more cautious if they knew such things were going to happen... All of this occurs because of parents’ lack of awareness.” [Rita, Health Volunteers group]*

Mothers were aware of child injury events because it had happened to their children, but they had no idea how to improve home safety.

*“Some mothers are unaware that if they leave certain items in one location, the children will play with them, causing accidents. There is a dearth of understanding among mothers.” [Lalita, Mothers focus group]*

Many participants suggested that raising awareness through educational programme may help individuals recognise their duty to improve their living environment, for example:

*“... before, we conducted relatively few pregnancy tests. Due to a lack of knowledge, a small number of women came [to the health post] to give birth. MIRA launched a public awareness campaign. Pregnant ladies now come for check-ups, form groups, and everything is well. That is necessary to keep in mind. This too will change, and far more quickly.” [Rita, Health Volunteers group]*

The student group also believed that training mothers would be a priority since they were the carers likely to spend the most time with their children and should be knowledgeable about the safety of their children. They also thought that people would pay more attention to training from an external organisation.

*“While students such as us may advise our parents to build the railings on the balcony, they may not follow our advice. If individuals from a particular institution or organisation say so, they will believe it.” [Hemant, Students focus group]*

### Motivation and affordability to reduce hazards

Participants across all groups were able to describe actions that would enhance the home safety. They thought that installing rails on the balcony, grills on the windows and fences surrounding water bodies would safeguard their children from falls and drowning.

*“The rich can afford to have proper grills installed on their doors and windows. Those of us who cannot afford it may utilise bamboo to create a safe environment for our children. A little piece of wood may be used to hang the sharp tools on the walls [the wood can be nailed to the wall and the sharp tools can be hooked behind it]. It is something that can be done.” [Rita, Health Volunteers group]*

Participants stated that the majority of homes in their area lacked adequate storage for hazardous materials. Many homes had few rooms, and some were single-room dwellings. As a result, several participants considered keeping harmful substances such as sharp instruments, poisons and chemicals out of reach of children as a possible option for injury prevention.

*“We should store the weapons in an inaccessible location. Glasses and sharp objects should be kept out of children’s reach.” [Susma, Health Volunteers group]*

Some teachers’ group participants believed that it was preferable to keep harmful substances out of sight rather than merely out of reach of children.

*“It is better to keep hazardous substances up high or in places where children cannot see them. Even keeping the substances in a higher position if they see they will try to reach. So, it’s always a good idea to keep hazardous substances out of children’s reach.” [Sarita, Teachers focus group]*

As the discussion progressed, participants shared examples of ways they or others in their community could improve safety. The majority of examples appeared to be related to the installation of safety devices and structural changes to their homes that they could afford and that utilised local resources.

Regarding the quality of built homes, many participants stated that their homes were unsuitable for children and that their children lived in substandard housing. Building a safe house in hilly areas is a difficult task due to the time and money required to flatten the land. The majority of their community’s residents were unable to afford the associated costs. As a result, they were obliged to build their home on a small plot of land with few rooms and without a separate kitchen or living room, which increases the risk of injury to children. Participants in the fathers’ and teachers’ groups expressed their views:

*“Poorly built houses require more money and effort to maintain or change.” [Hari, Fathers focus group]*

*“I really want to build an additional room to use a separate kitchen. But I cannot because the available space is insufficient.... On one side, there is a cliff; on the other, there is a risk of stones falling down the hill and into the house.” [Binita, Teachers focus group]*

Participants emphasised the need of resources and financial help in assisting certain individuals in changing their home environment, especially those who are financially weak. They stated that they were unable to afford both labour and essential materials due to their poor income.

*“There are some people who are struggling financially. They have a tiny piece of land and don’t even have enough food to eat. They may think that if they were given money or housing material, they would improve their housing situation... Regardless of their concern, they cannot just go and ask.” [Sabina, Health Volunteers group]*

### Cultural practices and shared responsibilities

In nearly all groups, close supervision of children has emerged as a consistent approach for preventing injury. The parents said they had some understanding of the risks of harm to their children whilst they work but do not have anyone else to watch over their child. Therefore, they tie their children to the furniture or building to keep them from accessing unsafe areas whilst they are alone. Parents thought that the children grow used to being tied up.

*“There is no one at home to care for our children when we go for work, and they [children] willingly allow their legs to be tied. This has become their habit (everyone laughed). We have seen it in other places of our village as well.” [Maya, Mothers focus group]*

Participants believed that child injury is a common problem in their community. Hence, ensuring that children are safe in their homes shouldn’t just be the responsibility of a few individuals or families, but should be shared by everyone in the community.

*“We should not focus only on keeping our homes safe, since children may visit other homes too. This pertains to our whole community... Every local neighbourhood should participate. We should educate our neighbours about such matters.” [Sunil, Fathers focus group]*

*“Like we are discussing here, if we could have similar discussions in our community, it would help parents understand how to improve their home safety... We could go to individual homes and inform them about child safety... We may discuss these things in the mothers’ group.” [Radhika, Health Volunteers group]*

The teachers believed that if one community could develop good injury prevention practices, it could be used as an example of how to make the home environment safe for children. They thought that other communities might be motivated to do the same.

## Discussion

This study explored participants’ views on the prevention of unintentional home injuries to pre-school children and identified both the potential for home environmental change at a community level and the barriers to, and facilitators of, such changes. The aim of the study was achieved by conducting focus group discussions in a variety of locations with diverse participants representing different groups in the community.

The most apparent and unambiguous message that emerged from all group discussions was that child injury is a prevalent issue in rural communities, but it is still largely ignored by parents and caregivers. Mothers appeared to be the least aware of possible injury risks compared to other group participants. This could be why participants across all groups believed that supporting mothers would be a priority because they were the caregivers most likely to spend the most time with their children. Except for mothers, participants in all groups were able to describe actions that would improve home safety. However, the affordability of improving home safety was primarily highlighted in the fathers’ group. In the mothers’ group, the issues related to inadequate child supervision and cultural practices for keeping children safe were mainly discussed. Participants, largely from the groups of health volunteers, fathers and teachers, emphasised the importance of the entire community taking responsibility for child safety.

Inadequate carer supervision was reported as a risk factor and has been found to be associated with higher incidence of child injuries (Morrongiello et al., 2004, 2011). The combination of parental (increased supervision and improved parents’ safety behaviours) and environmental (removing hazards and using safety equipment) strategies were proven as an effective way of reducing the frequency of childhood home injury (Morrongiello et al., 2004; Guilfoyle et al., 2012). This could explain why, without environmental changes, supervision or teaching strategies alone have been ineffective in Nepal in reducing child injury. While supervision may have prevented some injuries, not all parents in rural Nepal are able to provide their children with constant supervision. In low-income communities, parents’ economic activities, such as work, limit their ability to supervise their children (Munro et al., 2006). Participants revealed that some parents tie children’s legs to the furniture or building to keep them from accessing unsafe areas whilst they are alone. This suggests that there are few opportunities for parents to be supported with supervision of their children if they have to work and that culturally appropriate solutions need to be developed to support parents.

In the focus groups, safety education that could improve parents’ knowledge and skills to reduce injury hazards in their local environment was highlighted as an important strategy for child injury prevention. The findings corroborated those of a qualitative research conducted in Mexico, which recommended culturally appropriate home visits and environmental modification interventions as potential strategies for preventing unintentional injury in young children. (Crosslin and Tsai, 2016). Parents in low-income areas are unable to afford interventions that require a significant investment of time and resources. As a result, resource-intensive interventions are unlikely to be long-term sustainable. Environmental change interventions that are designed and implemented using low-cost, locally accessible resources and address community needs are more likely to be effective and sustainable (Munro et al., 2006; Rahman et al., 2010). This may be because low-income households prioritise food and living expenses above investing in home safety (Olsen et al., 2015).

While participants were able to propose a variety of possible ways for environmental change, it was unclear whether any members of the community had implemented such changes in their houses. A prior research in Nepal found that parents generally believed that warning or educating children about the risk of injury was the most essential strategy for injury prevention, and as a result, they put less effort into making hazardous environments safer for their children (Pant et al., 2014). Similar findings were reported in a study conducted in Bangladesh, where people in rural areas were generally aware of the causes and methods of child drowning prevention but rarely took preventative measures to keep their children safe from drowning (Rahman et al., 2008). In addition to belief and culture, a study from Iran revealed that parents with lack of money and time were unable to change their home environment (Barat et al., 2017).

In Nepal, there are no previous studies that have reported barriers and facilitators to preventing child injuries within the home. In high-income countries such as the UK, the main identified barriers to child injury prevention were lack of parental anticipation of injury-producing events, lack of knowledge about the consequences of injury and inappropriate timing of parental safety information that does not match the age and stage of development of their child (Ablewhite et al., 2015). A quantitative survey of parents in 14 European countries also reported that lack of awareness or knowledge about the causes of injury was a barrier to child injury prevention (Vincenten et al., 2005). In this regard, findings of reviews suggested that awareness programme and culturally sensitive information should be delivered to parents for enabling them to prevent their children from injury (Smithson et al., 2011; Ingram et al., 2012).

Findings from other countries may not be generalisable to the Nepalese context because the housing structures, family characteristics, living environment and cultural systems in the country are very distinct to those in HICs. In Nepal, the ideal injury prevention programme should include injury prevention education for parents in order to raise awareness of injury risks and empower parents to keep their homes safe. In addition to education, an intervention that enables parents to have informed discussions within their households and communities and lead to identification of affordable and achievable ways to make their homes safer is important.

### Strengths and limitations

The strength of this study was that this is the first study conducted in Nepal to explore community perceptions of home injuries that affect pre-school children, injury hazards in the home and the possibility of changes to the home environment to combat these. Two other researchers (TD and JM) read different transcripts independently to help develop a coding framework, reviewed the themes and associated codes (Noble and Smith, 2015) to reduce the subjectivity and enhance the credibility of the findings (Shenton, 2004). The study also had some limitations: as with all qualitative research, it is not possible to generalise these findings to a wider population. However, focus groups with people who represented key elements of the community, and in a variety of locations, helped to provide a broader representation of the views and experiences of the community. Findings from this qualitative study are likely to be transferrable to communities of people that live in similar situations. Only one focus group was conducted with each type of participant. Additional themes may have arisen if multiple focus groups were conducted with each type of participant.

### Implication for practice

In Nepal, there are no evidence-based programmes to support parents to keep their children safe in the home. These findings can be used to advocate for practice and policy change that builds a culture of child safety in communities and supports communities to take action to prevent child injuries in the home. FCHVs could use such information to stimulate discussion with mothers’ groups on child injuries and the management of home hazards. Similarly, healthcare workers at hospitals or local-health facilities could share the information with parents when they present with a pre-school child who has been injured. During antenatal care sessions, healthcare workers could also share the information with mothers or pregnant women. Being able to identify hazards and take action to address them may improve injury risk in communal areas of villages and benefit wider groups.

## Conclusion

This study identified several potential environmental changes that could help to reduce home injuries in young children and promoted understanding of factors that may help or hinder their implementation. Those responsible for developing home injury prevention interventions should consult with communities to identify environmental changes that are both locally and culturally relevant. There is a need for community-based educational interventions that support parents in rural Nepal to identify and reduce the number of home injury hazards for pre-school children. The study findings highlight the need for further qualitative research to understand why injuries are being accepted as a normal part of childhood and what would encourage people feel empowered to keep themselves and their children safe.

## Supplemental Material

sj-pdf-1-chc-10.1177_13674935211052156 – Supplemental Material for Home environmental change for child injury prevention in Nepal: A qualitative study Click here for additional data file. Supplemental Material, sj-pdf-1-chc-10.1177_13674935211052156 for Home environmental change for child injury prevention in Nepal: A qualitative study by Santosh Bhatta, Julie Mytton and Toity Deave in Journal of Child Health Care
