# Supplementary material for: Home environmental change for child injury prevention in Nepal: A qualitative study
Source: J Child Health Care. 2021 Nov 29;27(3):323–35. doi: 10.1177/13674935211052156 (PMC10472719; doi:10.1177/13674935211052156)
Supplement: sj-pdf-1-chc-10.1177_13674935211052156 – Supplemental Material for Home environmental change for child injury prevention in Nepal: A qualitative study [file sj-pdf-1-chc-10.1177_13674935211052156.pdf]

Supplemental material

Table S1. Common home injury hazards identified during the focus groups and quotes regarding incidents that led to fatal injuries in pre-school children

Injury mechanisms	Common hazards described by participants	Quotes (X is used to denote a person's name whilst keeping them anonymous). Pseudonyms have been used when reporting participant's quotes
Fall	Stairs/ladders and balconies without protective railings; narrow and slippery stairs; windows without bars; high porch or entrance area; moss on the floor around the water tap; falls from cradle (cot).	*There was one lady in our village who left her five months old daughter sleeping on a balcony that was not barred. The child fell from the balcony and died. [Renuka, Health Volunteers group]*
Drowning	Water in containers such as a bucket, jar or drum; ponds, wells and irrigation channels near the home.	*In the pond, above the hill, water is stored in a reservoir in a drum and children go there to fetch water. One child got inside the big drum saying he would swim and drowned. [Sarita, Teachers focus group]*<br>*Don't you remember X's daughter? While the mother was washing clothes, and fetching water from the well, the daughter climbed into the bucket and died. [Rita, Health Volunteers group]*<br>*We have heard that children fall into the drum filled with water and die. It has happened in the west. Usually, they keep water in huge jars. It is very hot there … In one incident, I don't know whether the lid of the jar was left open or something, but the kid drowned in the water. This was also in the news. [Hari, Fathers focus group]*
Fire, burn or scald	Open fire, matchsticks, or lighters; boiled water for domestic use; hot liquid food; acid kept for cleaning purposes.	*A child also died once. The clothes did not dry in the monsoon, so they kept them near the fire to dry. They also left the child by the fire to warm him up. The mother went to cut the grass after covering the child with a blanket. They do not even know when the fire caught. After she had returned from cutting the grass, the child was already dead. [Sabina, Health Volunteers group]*
Poisoning	Fertilizers, pesticides, insecticides and other household chemicals; medicine for the family or cattle.	*One or two years ago, a child died by taking pesticide…They [parents] were in the field putting pesticide amongst the corn. She [mother] had left the rest of the pesticide near her son. The son thought it was sugar and ate it. [Mina, Mothers focus group]*<br>*There was one incident when two children ate pesticide that was kept below the bedding after being used on the rice farm. They [children] saw it and ate it. When their father asked what they had eaten, they said they had eaten sugar which was stored below the bed. They died within two hours and before reaching the hospital …. One died immediately and one an hour later. They were brother and sister from the same family. [Kabita, Teachers focus group]*

Animal and insect related	Sting from honeybee; kicking or bites from domestic animals like cows, buffaloes, goats etc.	*Yes, they died. They died because a killer bee stung them. X's sister died due to bee stings at home. [Sabina, Health Volunteers group]*<br><br>*Once, a child was crying whilst the mother was trying to make him sleep. She was very tired so let the child sleep in the cradle and she slept on the bed beside it. The cat came and sat over the child and the child died …The cat came in search of warmth and it was warm in the cradle, so the cat sat on the child's face. He died of asphyxiation. [Rita, Health Volunteers group]*
Suffocation or choking	Covering of the child's face with cloth to protect them from houseflies; plastic bags; small items such as food (e.g., corn and beans), coins and marbles.	*Also, whilst breast-feeding the child, its mother fell asleep and the child died because her breasts pressed over the child's face. [Monika, Health Volunteers group]*<br><br>*In our village, also whilst breast-feeding at night, the mothers' weight on her daughter [after falling asleep] suffocated the child who then died. [Renuka, Health Volunteers group]*<br><br>*It occurs when mothers breastfeed their children... My own nephew died. My nephew latched on to breastfeed and my sister-in-law fell asleep … he died as the breast suffocated him. [Kopila, Teachers focus group]*
